# Avalanches in Self-Organized Critical Neural Networks: A Minimal Model for the Neural SOC Universality Class

**DOI:** 10.1371/journal.pone.0093090

**Published:** 2014-04-17

**Authors:** Matthias Rybarsch, Stefan Bornholdt

**Affiliations:** Institut für Theoretische Physik, Universität Bremen, Bremen, Germany; National Research & Technology Council, Argentina

## Abstract

The brain keeps its overall dynamics in a corridor of intermediate activity and it has been a long standing question what possible mechanism could achieve this task. Mechanisms from the field of statistical physics have long been suggesting that this homeostasis of brain activity could occur even without a central regulator, via self-organization on the level of neurons and their interactions, alone. Such physical mechanisms from the class of self-organized criticality exhibit characteristic dynamical signatures, similar to seismic activity related to earthquakes. Measurements of cortex rest activity showed first signs of dynamical signatures potentially pointing to self-organized critical dynamics in the brain. Indeed, recent more accurate measurements allowed for a detailed comparison with scaling theory of non-equilibrium critical phenomena, proving the existence of criticality in cortex dynamics. We here compare this new evaluation of cortex activity data to the predictions of the earliest physics spin model of self-organized critical neural networks. We find that the model matches with the recent experimental data and its interpretation in terms of dynamical signatures for criticality in the brain. The combination of signatures for criticality, power law distributions of avalanche sizes and durations, as well as a specific scaling relationship between anomalous exponents, defines a universality class characteristic of the particular critical phenomenon observed in the neural experiments. Thus the model is a candidate for a minimal model of a self-organized critical adaptive network for the universality class of neural criticality. As a prototype model, it provides the background for models that may include more biological details, yet share the same universality class characteristic of the homeostasis of activity in the brain.

## Introduction

Information processing by a network of dynamical elements is a delicate matter: Avalanches of activity can die out if the network is not connected enough or if the elements are not sensitive enough; on the other hand, activity avalanches can grow and spread over the entire network and override information processing, as e.g. observed in epilepsy. Therefore, it has long been argued that neural networks have to establish and maintain a certain intermediate level of activity in order to keep away from, both, the regimes of chaos and silence [1], [3]–[5]. Similar ideas were also formulated in the context of genetic networks where Kauffman postulated that information processing in these evolved biochemical networks would be optimal near the “edge of chaos”, or the critical regime of the dynamical percolation transition of such networks [6].

In the wake of the discovery of self-organized criticality (SOC) it was asked if also neural systems were self-organized to some form of criticality [7]. An early example of a SOC model that had been adapted to be applicable to neural networks is the model by Eurich et al. [8]. Their model is a variant of the random neighbor Olami-Feder-Christensen model for earthquakes and exhibits, subject to one critical coupling parameter, distributions of avalanche sizes and durations which they postulate could also occur in neural systems.

Another early example is a spin model for self-organized critical neural networks [1], [9] that draws on the alternative approach of self-organized critical adaptive networks [10]. Here networks are able to self-regulate towards and maintain a critical system state, via simple local rewiring rules which are plausible in the biological context.

Only after these first hypothetical models, experimental evidence for criticality in neural systems has been found in terms of spatio-temporal activity avalanches, first in the seminal work of Beggs and Plenz [2]. Much further experimental evidence has been collected since, which we will briefly review below. Only recently, however, experimental data has reached the resolution to discuss the hypothesis of dynamical criticality in neural tissue in the context of measurements. A major finding is that these new data match well with scaling theory of non-equilibrium critical phenomena, providing us with a solid evidence for criticality in cortex tissue dynamics [22]. As a result this sheds new light on the early spin models of self-organized critical adaptive neural networks, where now their predictions can actually be tested against the new observations. This is the purpose of this paper.

The outline of this paper is as follows. We will first briefly review the further experiments on neural activity avalanches. Then we will give an overview of models that have been motivated by these observations. We will then revisit the earliest spin model for self-organized critical adaptive neural networks [1] and test its applicability in the light of experimental data. We redefine the model for a natural representation of activity avalanches [11], study the avalanche dynamics of the model, and discuss its relation to criticality in the context of the scaling theory of non-equilibrium critical phenomena.

### Avalanche dynamics in neuronal systems

Let us first briefly review the experimental studies on neuronal avalanche dynamics. In 2003, Beggs and Plenz published their findings about a novel mode of activity in neocortical neuron circuits [2]. During *in-vitro* experiments with cortex slice cultures of the rat, they found evidence of spontaneous bursts and avalanche-like propagation of activity followed by silent periods of various lengths. The observed power-law distribution of event sizes indicates that the neuronal network is maintained in a critical state. Also, the spatio-temporal patterns of the avalanches are stable and precise over many hours and robust against external perturbations [12], which indicates that they might play a central role for brain functions as, for example, information storage and processing. Neuronal avalanches have also been found during developmental stages of *in-vitro* cortex slice cultures from newborn rats [13], as well as in cultures of dissociated neurons in different kinds of networks, as rat hippocampal neurons and leech ganglia [14], or rat embryos [15].

Aside from these *in-vitro* experiments, extensive studies *in-vivo* have since been conducted. The emergence of spontaneous neuronal avalanches has been shown in anaesthesized rats during cortical development [16] as well as in awake rhesus monkeys during ongoing cortical synchronization [17].

The biological relevance of the avalanche-like propagation of activity in conjunction with a critical state of the neuronal network has been emphasized in several works recently. Such network activity has proven to be optimal for maximum dynamical range [18], [19], maximal information capacity and transmission capability [20], as well as for a maximal variability of phase synchronization [21]. Most recently, experimental evidence for universality of critical dynamics has been found in neuronal avalanche data [22]–[24] and formally linked to universal scaling theory [25]. This can be considered as providing a solid evidence for dynamical criticality in neuronal systems.

### Models for neural criticality

These experimental studies with their rich phenomenology sparked a large number of theoretical studies and models for criticality and self-organization in neural networks, ranging from simple toy models to detailed representations of biological functions.

A variety of models have been constructed that are careful to include biological details at the neuron level as a basis for possible self-organization. Such mechanisms include threshold firing dynamics and activity-dependent plasticity of synaptic couplings as the basis for self-organization. While some models feature synaptic facilitation following a firing event [26]–[28], others use synaptic depression as the main driving force towards criticality [29], [30]. It has been shown that anti-Hebbian evolution is generally capable of creating a dynamically critical network when the anti-Hebbian rule affects only symmetric components of the connectivity matrix, while the anti-symmetric component remains as an independent degree of freedom utilizable for e.g. learning tasks [31]. Also, synaptic plasticity on two different timescales has been discussed [32].

On the other hand, the biological plausibility of activity-dependent synaptic plasticity for adaptive self-organized critical networks has been emphasized [33]. Recently, correlations of subsequent firing events again came into focus as a synaptic facilitation criterion [34]. The biological relevance of the critical state in neural networks for a brain function as learning has further been underlined [35]. Most recently, the temporal organization of neuronal avalanches in real cortical networks has been linked to the existence of alternating states of high vs. low activity in the network as well as to a balance of excitation and inhibition in a critical network [36].

While the proposed organizational mechanisms strongly differ between the individual models, there are signs that the resulting evolved critical networks may be part of the same fundamental universality class. Many of the models exhibit at least some of the avalanche statistics seen in the experimental data, as e.g. a power-law distribution with exponent around 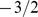 for the distribution of avalanche sizes. With the recent, more detailed models in mind, we are especially interested in the underlying universality of self-organization.

### Revisiting the spin model of self-organized critical neural networks

Let us now revisit the earliest spin models of self-organized critical neural networks [1], [10] in a formulation that allows for studying its avalanche dynamics in time and space. Two main aspects have to be addressed.

First, the spin-type description of the dynamical variables, due to its symmetrized nature, does not allow to sample avalanche statistics at the critical point. We therefore translate the model into a version with Boolean state nodes and redefine its activation threshold function and its network rewiring mechanism accordingly. As a result, activity avalanches intrinsically occur in the network, whereas spin networks typically exhibit continuous fluctuations with no avalanches directly visible. The further advantages of this transformation in the context of biological networks have been discussed in a previous paper [11].

The second aspect to be reviewed is the topology the algorithm operates on. While the original correlation-based rewiring mechanism of network self-organization [1] has been defined to simply operate on neighboring nodes on a lattice, we would like to study the model here as an arbitrary self-organizing network, without specifying any underlying topology. However, while on a lattice the number of possible neighbors of a node is strictly limited, on a large random network near critical connectivity there are far more unconnected pairs of nodes than there are connected pairs. Thus, randomly selecting pairs of nodes for rewiring would introduce a strong bias towards connecting nodes which were previously unconnected. This bias would result in a strong increase of connectivity, far above any self-organized critical regime. Consequently, we will adapt the rewiring mechanism below to include arbitrary topologies without such a bias.

The philosophy of the model is its capability of self-regulation towards a critical state despite being simplified to the most minimal model possible. Its rewiring mechanism is based on a simple rewiring rule, which only uses information accessible to individual nodes locally, which here means pre- and post-synaptic activities of the particular node, as well as correlations of these activities.

## Methods

### Adaptive network evolution

We will now first define the dynamics *on* the network and will then proceed with the rewiring dynamics, i.e. the dynamics *of* the network.

Consider a randomly connected network of 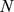 nodes of Boolean states 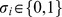 which can be linked by asymmetric directed couplings 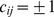. Node pairs which are not linked have their coupling set to 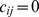. Links may exist between any two nodes, so there is no underlying spatial topology in this model. Let 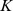 denote the average connectivity of the network, i.e. the number of in-links averaged over all 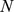 nodes.

All nodes are updated synchronously in discrete time steps via a simple threshold function of their input signals with a small thermal noise introduced by the inverse temperature 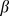 in the same way as in the original version of the model [1]. However, now an input shift of 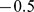 adds to the Glauber update, representing the modified update function in the course of the transition from spins to Boolean node values [11]:
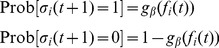
(1)with
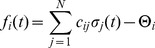
(2)and
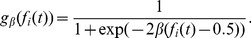
(3)


For simplicity, we assume that all nodes have an identical activation threshold of 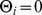, unless stated otherwise.

### Rewiring algorithm

The correlation-based rewiring mechanism of the original model [1] has to be carefully revised as well, when changing from spin variables to Boolean variables, as inactive nodes are now represented by a value of 0 instead of −1 which affects the calculation of correlations.

The adaptation algorithm operates as follows. After initializing the network with random links at a given initial connectivity 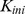 and initial states set to 0, all nodes are synchronously updated in parallel according to eq. (1). All activity then observed in this model originates from small perturbations by thermal noise, leading to activity avalanches of various sizes. In the following we set the inverse temperature to 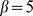. On a larger time scale, here after 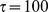 updates, a rewiring is introduced at one randomly chosen, single node. The new element in our revised model is to test whether the addition or removal of one random in-link at the selected node will increase the average dynamical correlation to all existing inputs of that node. By selecting only one single node for this procedure, we effectively diminish the bias of selecting mostly unconnected node pairs – but retain the biologically inspired idea for a Hebbian, correlation-based rewiring mechanism on the basis of locally available information, only.

Now, we have to define what is meant by *dynamical correlation* in this case. We here use the Pearson correlation coefficient to first determine the correlation between a node *i* and one of its inputs *j* over the preceding 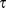 time steps:
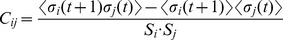
(4)where 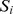 and 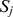 in the denominator denote the standard deviations of the states of nodes *i* and *j* respectively. In case one or both of the nodes remain frozen in their state (i.e. yield a standard deviation of 0), we will assume a correlation of 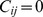, as otherwise the Pearson correlation coefficient would not be well defined.

Note that we always use the state of node *i* at one time step later than node *j*, thereby taking into account the signal transmission time of one time step from one node to the next one. Finally, we define the average input correlation 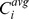 of node *i* as
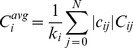
(5)where 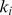 is the in-degree of node *i*. The factor 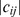 ensures that correlations are only measured where links are present between the nodes. For nodes without any in-links (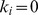) we define 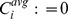.

In detail, the adaptive rewiring is now performed in the following steps:

1. Select a random node *i* at which the next rewiring will take place.
2. Run network updates for 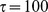 simulation time steps and measure the average input correlation 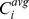 of node *i*.
3. With equal probability, either
  - insert an additional in-link of random weight 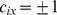 at node *i* from a random, previously unlinked node *x*, or
  - remove one of the existing in-links at node *i*.
4. Again run 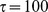 network updates and measure the new 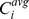 of node *i* after the local rewiring at this node.
5. If 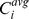 has increased after the insertion or removal of the in-link, the rewiring from step 3 is retained; otherwise, it is reverted.
6. Run 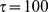 network updates to allow for a transient period prior to the next rewiring process. Iterate from step 1.

Note that the exact choice of 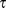 is not critical, but is chosen as 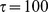 here to ensure time scale separation of at least two orders of magnitude between node dynamics (fast) and rewiring changes (slow).

It is also worth noting that this updated model version – in the same way as the original model [1] – is solely based on locally available information at synapse level and takes into account both pre- and post-synaptic activity. This is a fundamental difference to approaches discussed e.g. in [26], [27] or [29], where only pre-synaptic activity determines changes to the coupling weights.

In order to obtain an indication of the current dynamical regime of the network (i.e. whether the network is sub- or super-critical, or close to the critical point), we continuously measure a branching parameter based on potential damage spreading in the network. This is realized by counting, for each individual node *i*, the number of descendant nodes which would possibly change their states at time step 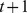 if the state of node *i* was changed at the present time *t*. Here, both, the present states (on or off) of node *i* and its descendants, as well as the nature of their respective links (activating or inhibiting) are taken into account. The obtained number of descendant nodes prone to damage spreading (and thus also to signal propagation) is then averaged over the entire network, resulting in the branching parameter 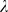. This allows to estimate (based on the current network configuration) whether the network is sub-critical (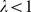) or super-critical (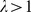). For the analysis of avalanche statistics in the evolved critical networks we export snapshots of the network structure whenever the branching parameter is close to one (here when 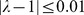).

### Avalanche analysis

For a detailed analysis of avalanche statistics, we use the snapshots of near-critical (per branching parameter estimation) network structures from the adaptation runs as outlined above. Key observables are the avalanche size 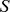, i.e. the total number of nodes which become active at least once during one avalanche, and the avalanche duration 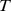, i.e. the number of simulation time steps from the start (first node active) to the termination (no more nodes active) of an avalanche. To obtain those, the network is now run in a deterministic mode with 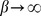. The update function from (1) then simplifies to
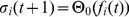
(6)with a redefined threshold function (nodes only become active with activating, non-zero input)
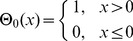
(7)and the usual input function
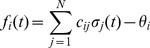
(8)where activation thresholds 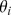 are set to 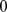 for now. With parallel updates, any network activity would eventually end up in either a fixed point or limit cycle attractor, but not necessarily at the fixed point “all nodes off”, terminating an avalanche. Therefore, we introduce an exhaust time parameter 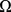 which can be biologically interpreted as an effect of depleting neuro-transmitters at active synapses. In each time step, every node will increase its individual activation threshold 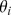 to 1 with a probability corresponding to its own average activity over the last 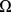 time steps (i.e. number of time steps where 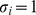 divided by the total number of time steps 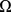). This turns out to be sufficient to eventually step out of a periodic attractor and terminate the avalanche. Whenever one avalanche is terminated (all nodes off), we will start a new one by randomly activating one single node and continue with the parallel updates. We constantly keep track of cumulative avalanche size and duration distributions, 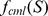 and 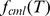, as well the average size 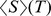 of avalanches that have a certain duration 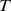. From universal scaling theory [25] we expect the following power-law scaling relations in case of critical networks:
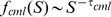
(9)

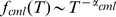
(10)

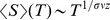
(11)where the exponents fulfill
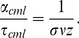
(12)


## Results

### Adaptive network evolution

In the following, we will have a look at different observables during numerical simulations of network evolution in the model. Key figures include the average connectivity 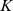 and the branching parameter 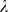. Both are closely linked to, and influenced by, the ratio of activating links 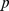 which is simply the fraction of positive couplings 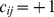 between all existing (non-zero) links.

**Figure 1:**
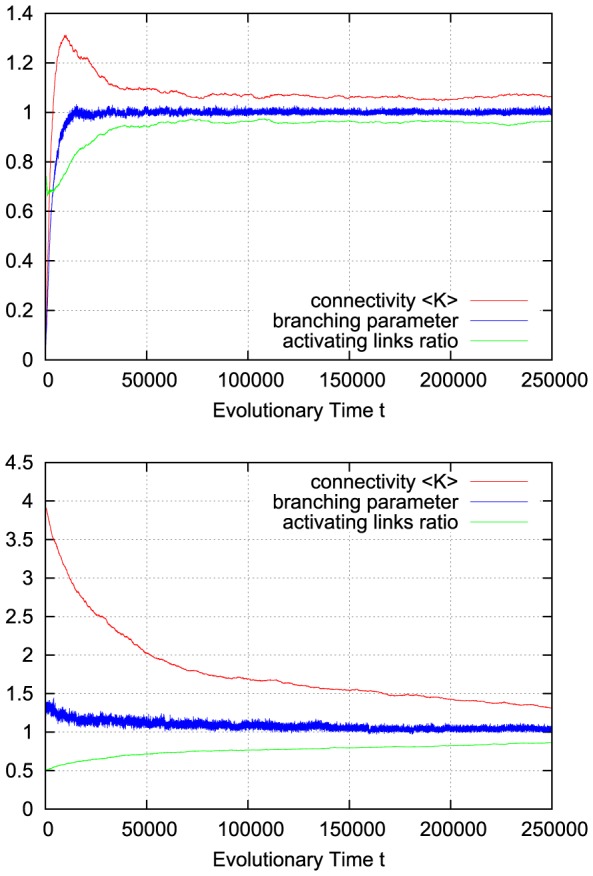
Typical run of the network self-organization algorithm. Regardless of initial connectivity and dynamical regime, the network evolves to a critical configuration. Top: when starting with completely isolated nodes the “network” is obviously subcritical and links will be inserted. Thus, both the connectivity (red) and the branching parameter (blue) as an indicator of network criticality increase. The network approaches a critical state where the branching parameter stabilizes close to one. Bottom: with higher initial connectivity, the network is supercritical at first. Links are removed from the network while the branching parameter approaches the critical value of one. As the self-organization algorithm is constructed to maximize activity correlations between linked nodes, the ratio of activating links (green) slowly increases in both cases.

The upper part in Figure 1 shows a typical run of the topology evolution algorithm, where we start with completely isolated nodes without any links. Trivially, the “network” is subcritical at this stage, which can be seen from the branching parameter which is far below 1. As soon as rewiring takes place, the network starts to insert new links, obviously because these links enable the nodes to pass signals and subsequently act in a correlated way. With increasing connectivity, also the branching parameter rises, indicating that perturbations start to spread from their origin to other nodes. When the branching parameter approaches 1, indicating that the network reaches a critical state, the insertion of new links is cut back. The processes of insertion and depletion of links tend to be balanced against each other, regulating the network close to criticality.

On the other hand, if we start with a randomly interconnected network at a higher connectivity as, for example, 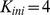 (see lower part of Figure 1), we find the network in the supercritical regime (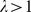) at the beginning. When above the critical threshold, many nodes will show chaotic activity with low average correlation to their respective inputs. The rewiring algorithm reacts by deleting links from the network, until the branching parameter approaches 1.

In both examples above, the evolution of the ratio of activating links 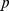 (which tends towards 1) shows, that the rewiring algorithm in general favors the insertion of activating links and vice versa the deletion of inhibitory couplings. Indeed, this appears quite plausible when we remind ourselves that the rewiring mechanism optimizes the inputs of a node towards high correlation on average. Also, nodes will only switch to active state 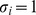 if they get an overall positive input. As we had replaced spins by Boolean state nodes, this can only happen via activating links – and that is why correlations mainly originate from positive couplings in our model. As a result, we observe the connectivity evolving towards one in-link per node, with the ratio of positive links also approaching one.

**Figure 2:**
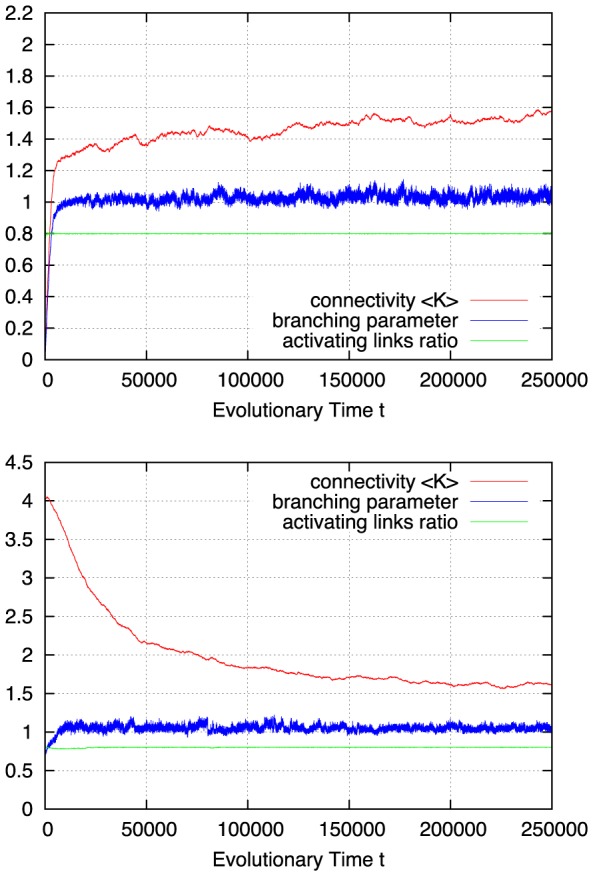
Typical run with fixed ratio of activating links. If the ratio of activating links (green) is kept fixed (here: 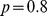; i.e. 80% activating links) in order to keep some inhibiting links within the network, the connectivity (red) evolves to a higher value. Still, the branching parameter (green) is maintained close to the critical value of one. Top: starting with isolated nodes (subcritical). Bottom: starting at supercritical connectivity.

For a richer pattern complexity, we might later want to introduce a second mechanism which balances out positive and negative links, and with a first approach we can already test how the rewiring strategy would fit to that situation: if, after each rewiring step, we change the sign of single random links as necessary to obtain a ratio of e.g. 80% activating links (i.e. 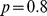), keeping the large majority of present links unchanged, the branching parameter will again stabilize close to the critical transition, while the connectivity is maintained at a higher value. Figure 2 shows that the self-organization behavior is again independent from the initial conditions. This result does not depend on the exact choice of the activating links ratio 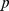; similar plots can easily be obtained for a large range starting at 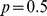, where the connectivity will subsequently evolve towards a value slightly below 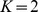, which is the critical connectivity for a randomly wired network with balanced link ratio according to the calculations made for the basic network model [11].

### Avalanche properties

**Figure 3:**
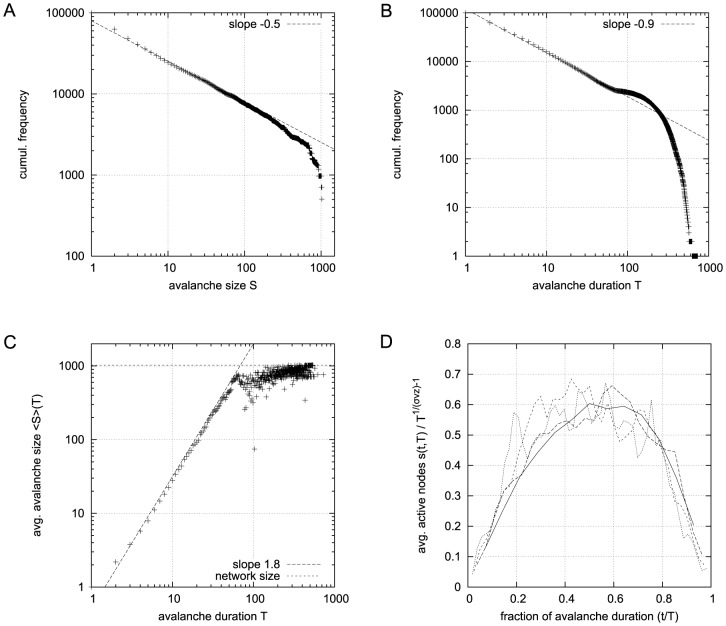
Critical exponents and collapse of avalanche profiles. Results measured from 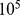 avalanches on 10 different evolved sample networks of 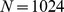 nodes, with an exhaust time parameter 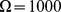. Similar results were gained with 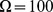 or 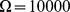, this choice has no significant effect on the scaling exponents. A: Cumulative distribution of avalanche sizes shows a power-law scaling 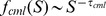 with exponent 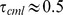. B: Cumulative distribution of avalanche sizes shows a power-law scaling 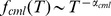 with exponent 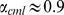. C: Average size 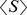 of avalanches of duration 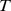 shows a power-law increase corresponding to 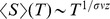 with an exponent of 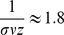. Note that the exponents 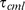, 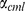, 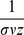 fulfill the relation 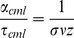 which is expected for a critical system. D: Profiles of avalanches, i.e. average scaled size as a function of scaled avalanche duration, of different durations (shown here for 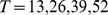) approximately collapse onto a universal shape.

In addition to the branching parameter measurement, let us now take a look at some dynamical properties of the evolved networks to further characterize their criticality. Figure 3 shows the distributions of avalanche size and duration, as well as further scaling properties. (A): The avalanche size 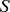 exhibits a power-law scaling 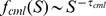 almost up to network size with an exponent 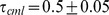 in the cumulative distribution, corresponding to a probability density exponent of 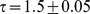. (B): Similarly, avalanche durations 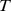 are power-law distributed as well up to a duration of approximately 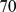 time steps, according to 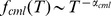 with an exponent of 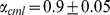, i.e. 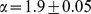. As discussed above, plain power-law distributions can originate from several mechanisms and cannot be considered alone as clear evidence of criticality. To obtain a third exponent, we have also measured the average avalanche sizes 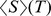 as a function of avalanche duration 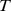. It becomes clear from (C) that for durations of approximately 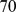 time steps and more, the avalanches begin to span most of the system size, which explains the cutoff position in the avalanche duration scaling (B). Up to that point, we find a power-law scaling 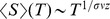 with an exponent of 
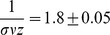
. These exponents are both in line with experimental results [22] and fulfill the exponent relation 
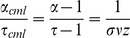
 as predicted for a critical system by the scaling theory of non-equilibrium critical phenomena [25]. (D): Finally, we find that avalanche profiles (i.e. average scaled size as a function of scaled avalanche duration) of avalanches of different durations 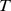 approximately collapse onto a universal shape, another feature of criticality also seen in the neural experiments [22].

### Variations in activation thresholds and response to external perturbation

**Figure 4:**
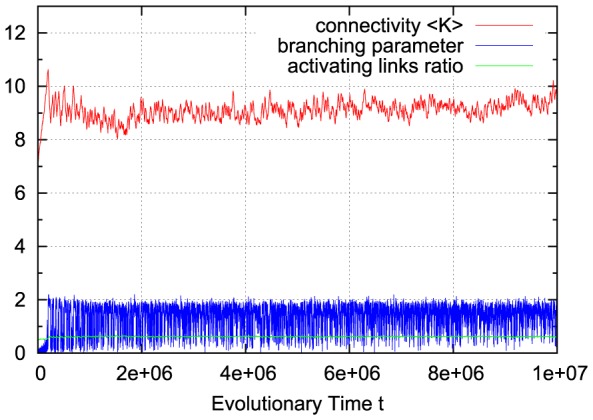
Typical run with higher activation thresholds 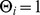. When activation thresholds are increased, a node needs more than one excitatory input to become active itself. Thus, higher overall connectivity is needed to allow signal propagation on a critical level. The adaptation process responds accordingly and maintains a connectivity around 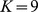 while the branching parameter shows larger fluctuations between sub- and supercritical states, but in general is kept on a moderate level and does not diverge with increasing connectivity.

In the above simulations, the activation thresholds of all nodes were strictly set to 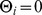 for maximum model simplicity. However, a neuron might as well need higher input to become active. Figure 4 demonstrates that the proposed adaptation algorithm similarly works well on networks of nodes with a non-zero activation threshold of e.g. 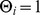. According to the update rule (1), now at least two positive inputs are necessary to activate a single node. As the rewiring algorithm is based on propagation of thermal noise signals, the inverse temperature 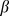 needs to be selected at a lower value than before. (As a general rule, 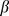 should be selected in a range where thermal activation of nodes occurs at a low rate, such that avalanches can be triggered, but are not dominated by noise.) The simulation is now started at an average connectivity of 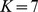 which is still sub-critical in this case (branching parameter low). In a similar way as shown above, the network adapts by inserting new links and increasing 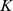, thereby also increasing the average branching parameter. While the system does not approach to a phase transition as nicely as shown above for activation thresholds of zero (in fact the branching fluctuates much more around the target value of one), the general tendency remains: the rewiring mechanism reacts properly before the network drifts too far off from criticality. The connectivity also fluctuates more, but stabilizes on a level around 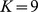.

In their *in-vitro* experiments, Beggs and Plenz further demonstrate that cortical networks can self-regulate in response to external influences, retaining their functionality while avalanche-like dynamics persist – for example after neuronal excitability has been increased by adding stimulant substances to the cultures.

**Figure 5:**
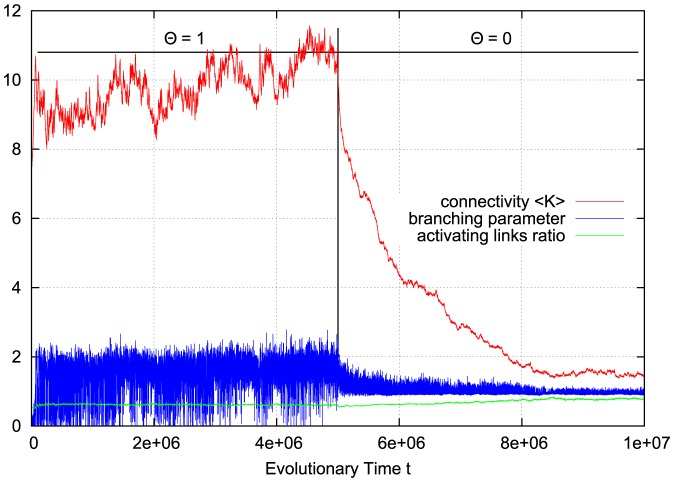
Rewiring response to a sudden decrease of activation thresholds. If we first set all node activation thresholds to 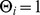, the connectivity (red) must first evolve to a higher value to allow propagation of activity within the network. When all activation thresholds are suddenly reduced (to mimic an external influence of neuronal excitability by stimulant substances) at the same time step, the network properly responds to the new situation and reduces connectivity to decrease excitability back to a critical level.

To reproduce such behavior in our model, we can include variations in the activation thresholds 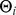 of the individual nodes. Assume we start our network evolution algorithm with a moderately connected, but subcritical network, where all nodes have an activation threshold of 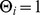. Figure 5 shows that the network first behaves in the same way as demonstrated in Figure 4 for activation thresholds 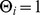. At one time step in the center of Figure 5, we at once reset all nodes to an activation threshold of 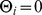, simulating the addition of a stimulant. As we can expect, this immediately puts the network into a supercritical, chaotic state. This is reflected by the branching parameter, which now constantly stays above one and does not fluctuate below anymore. It is clearly visible that the rewiring mechanism promptly reacts and drives back the connectivity, until the branching parameter slowly converges towards one again. A similar behavior is also found if thresholds 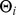 are not changed all at once, but gradually during network evolution.

## Summary and Discussion

To conclude, we have demonstrated that a very minimalistic binary neural network model is capable of self-organized critical behavior that matches the experimentally observed criticality in neural systems.

We revisited the earliest spin model for self-organized critical neural networks and transformed it to networks of nodes with Boolean node states and with arbitrary topology. The adaptive dynamics of the network is a simple, locally realizable rewiring mechanism which uses activity correlation as its regulation criterion, thus retaining the biologically inspired rewiring basis from the spin version of the original model. As a result the dynamical network exhibits emerging activity avalanches with spatio-temporal properties comparable to those observed in real neuronal systems.

What the model does not provide are hypotheses about possible details of implementations on the biological level. We did not make particular efforts to implement a fully local version, although such local, continuously running versions of the algorithm are straightforward. Instead we kept the stepwise procedure of separate correlation measurements at two different times for clarity. A biological implementation, in one form or another, has to sense the time derivative of the correlation for which there are numerous possibilities. Apart from that central detail it is obvious that on the local level there are far more details possible in a biological realization – some of which are contained in other existing models reviewed above – which we do not further discuss here. However, the central properties of criticality will be independent of these details. For future work, it might be fruitful to study particular biological realizations of the correlation based adaptation which we here studied in a bare bones algorithmic version. Further, it will be interesting to compare our algorithm with certain other models for neural adaptation with particular attention to the scaling properties at criticality.

In summary we find that the earliest spin model of neural criticality exhibits avalanche statistics that compare well with experimental data without the need for parameter tuning. The model represents a fundamental organization mechanism leading to a critical system that may serve as the simplest representative of a “neural SOC universality class”, matching the observed characteristics of self-organized criticality in biological cortical tissue. In particular, the model exhibits a scaling of avalanche size and duration distributions, as well as a universal scaling of the temporal avalanche profiles, altogether constituting the specific characteristics of neuronal avalanches near criticality.

## References

[1] Bornholdt S, Röhl T (2003) Self-organized critical neural networks. Phys Rev E 67: 066118. 10.1103/PhysRevE.67.066118 16241315

[2] Beggs JM, Plenz D (2003) Neuronal avalanches in neocortical circuits. J Neurosci 23: 11167–11177. 14657176 10.1523/JNEUROSCI.23-35-11167.2003 PMC6741045

[3] Langton CG (1990) Computation at the edge of chaos. Physica D 42: 12–37.

[4] Herz AVM, Hopfield JJ (1995) Earthquake cycles and neural reverberations: Collective oscillations in systems with pulse-coupled threshold elements. Phys Rev Lett 75: 1222–1225. 10060236 10.1103/PhysRevLett.75.1222

[5] Bak P, Chialvo DR (2001) Adaptive learning by extremal dynamics and negative feedback. Phys Rev E 63: 031912. 10.1103/PhysRevE.63.031912 11308683

[6] Kauffman S (1993) The Origins of Order: self-organization and selection in evolution. Oxford University Press.

[7] Bak P, Tang C, Wiesenfeld K (1988) Self-organized criticality. Phys Rev A 38: 364. 10.1103/physreva.38.364 9900174

[8] Eurich CW, Herrmann JM, Ernst UA (2002) Finite-size effects of avalance dynamics. Phys Rev E 66: 066137. 10.1103/PhysRevE.66.066137 12513377

[9] Bornholdt S, Röhl T (2001) Self-organized critical neural networks. arXiv:cond-mat/0109256v1. 10.1103/PhysRevE.67.066118 16241315

[10] Bornholdt S, Rohlf T (2000) Topological evolution of dynamical networks: Global criticality from local dynamics. Phys Rev Lett 84: 6114–6117. 10991137 10.1103/PhysRevLett.84.6114

[11] Rybarsch M, Bornholdt S (2012) Binary threshold networks as a natural null model for biological networks. Phys Rev E 86: 026114. 10.1103/PhysRevE.86.026114 23005832

[12] Beggs JM, Plenz D (2004) Neuronal avalanches are diverse and precise activity patterns that are stable for many hours in cortical slice cultures. J Neurosci 24: 5216–5229. 15175392 10.1523/JNEUROSCI.0540-04.2004 PMC6729198

[13] Stewart CV, Plenz D (2008) Homeostasis of neuronal avalanches during postnatal cortex development in vitro. J Neurosci Meth 169: 405–416. 10.1016/j.jneumeth.2007.10.021 PMC2743406 18082894

[14] Mazzoni A, Broccard FD, Garcia-Perez E, Bonifazi P, Ruaro ME, et al (2007) On the dynamics of the spontaneous activity in neuronal networks. PLoS ONE 5: e439. 10.1371/journal.pone.0000439 PMC1857824 17502919

[15] Pasquale V, Massobrio P, Bologna LL, Chiappalone M, Martinoia S (2008) Self-organization and neuronal avalanches in networks of dissociated cortical neurons. Neurosci 153: 1354–1369. 10.1016/j.neuroscience.2008.03.050 18448256

[16] Gireesh ED, Plenz D (2008) Neuronal avalanches organize as nested theta- and beta/gamma-oscillations during development of cortical layer 2/3. PNAS 105: 7576–7581. 18499802 10.1073/pnas.0800537105 PMC2396689

[17] Petermann T, Thiagarajan TC, Lebedev MA, Nicolelis MAL, Chialvo DR, et al (2009) Spontaneous cortical activity in awake monkeys composed of neuronal avalanches. PNAS 106: 15921–15926. 19717463 10.1073/pnas.0904089106 PMC2732708

[18] Shew WL, Yang H, Petermann T, Roy R, Plenz D (2009) Neuronal avalanches imply maximum dynamic range in cortical networks at criticality. J Neurosci 29: 15595–15600. 20007483 10.1523/JNEUROSCI.3864-09.2009 PMC3862241

[19] Kinouchi O, Copelli M (2006) Optimal dynamical range of excitable networks at criticality. Nature Physics 2: 348–351.

[20] Shew WL, Yang H, Yu S, Roy R, Plenz D (2011) Information capacity and transmission are maximized in balanced cortical networks with neuronal avalanches. J Neurosci 31: 55–63. 21209189 10.1523/JNEUROSCI.4637-10.2011 PMC3082868

[21] Yang H, Shew WL, Roy R, Plenz D (2012) Maximal variability of phase synchrony in cortical networks with neuronal avalanches. J Neurosci 32: 1061–1072. 22262904 10.1523/JNEUROSCI.2771-11.2012 PMC3319677

[22] Friedman N, Ito S, Brinkman BAW, Shimono M, DeVille REL, et al (2012) Universal critical dynamics in high resolution neuronal avalanche data. Phys Rev Lett 108: 208102. 23003192 10.1103/PhysRevLett.108.208102

[23] Priesemann V, Valderrama M, Wibral M, Le Van Quyen M (2013) Neuronal Avalanches Differ from Wakefulness to Deep Sleep - Evidence from Intracranial Depth Recordings in Humans. PLoS Comput Biol 9 (3) e1002985 doi:10.1371/journal.pcbi.1002985 23555220 10.1371/journal.pcbi.1002985 PMC3605058

[24] Scarpetta S, de Candia A (2013) Neural Avalanches at the Critical Point between Replay and Non-Replay of Spatiotemporal Patterns. PLoS ONE 8 (6) e64162 doi:10.1371/journal.pone.0064162 23840301 10.1371/journal.pone.0064162 PMC3688722

[25] Sethna JP, Dahmen KA, Myers CR (2001) Crackling noise. Nature 410: 242–250. 11258379 10.1038/35065675

[26] de Arcangelis L, Perrone-Capano C, Herrmann HJ (2006) Self-organized criticality model for brain plasticity. Phys Rev Lett 96: 028107. 16486652 10.1103/PhysRevLett.96.028107

[27] Pellegrini GL, de Arcangelis L, Herrmann HJ, Perrone-Capano C (2007) Activity-dependent neural network model on scale-free networks. Phys Rev E 76: 016107. 10.1103/PhysRevE.76.016107 17677533

[28] Meisel C, Gross T (2009) Adaptive self-organization in a realistic neural network model. Phys Rev E 80: 061917. 10.1103/PhysRevE.80.061917 20365200

[29] Levina A, Herrmann JM, Geisel T (2007) Dynamical synapses causing self-organized criticality in neural networks. Nature Physics 3: 857–860.

[30] Levina A, Herrmann JM, Geisel T (2009) Phase transitions towards criticality in a neural system with adaptive interactions. Phys Rev Lett 102: 118110. 19392248 10.1103/PhysRevLett.102.118110

[31] Magnasco MO, Piro O, Cecchi GA (2009) Self-tuned critical anti-hebbian networks. Phys Rev Lett 102: 258102. 19659122 10.1103/PhysRevLett.102.258102

[32] Peng J, Beggs JM (2013) Attaining and maintaining criticality in a neuronal network model. Physica A 392: 1611–1620.

[33] Droste F, Do AL, Gross T (2012) Analytical investigation of self-organized criticality in neural networks. J R Soc Interface 10: 20120558. 22977096 10.1098/rsif.2012.0558 PMC3565782

[34] de Arcangelis L, Herrmann HJ (2012) Activity-dependent neuronal model on complex networks. Front Physiol 3: 62. 22470347 10.3389/fphys.2012.00062 PMC3314197

[35] de Arcangelis L, Herrmann HJ (2010) Learning as a phenomenon occuring in a critical state. PNAS 107: 3977–3981. 20160107 10.1073/pnas.0912289107 PMC2840167

[36] Lombardi F, Herrmann HJ, Perrone-Capano C, Plenz D, de Arcangelis L (2012) The balance between excitation and inhibition controls the temporal organization of neuronal avalanches. Phys Rev Lett 108: 228703. 23003665 10.1103/PhysRevLett.108.228703

